# Oily Water Separation Process Using Hydrocyclone of Porous Membrane Wall: A Numerical Investigation

**DOI:** 10.3390/membranes11020079

**Published:** 2021-01-22

**Authors:** Sirlene A. Nunes, Hortência L. F. Magalhães, Ricardo S. Gomez, Anderson F. Vilela, Maria J. Figueiredo, Rosilda S. Santos, Fagno D. Rolim, Rodrigo A. A. Souza, Severino R. de Farias Neto, Antonio G. B. Lima

**Affiliations:** 1 Department of Fundamental and Social Sciences, Federal University of Paraiba, Areia, PB 58397-000, Brazil; sirlene.alves@academico.ufpb.br; 2 Department of Chemical Engineering, Federal University of Campina Grande, Campina Grande, PB 58429-900, Brazil; severino.rodrigues@professor.ufcg.edu.br; 3 Department of Mechanical Engineering, Federal University of Campina Grande, Campina Grande, PB 58429-900, Brazil; ricardosoaresgomez@gmail.com (R.S.G.); antonio.gilson@ufcg.edu.br (A.G.B.L.); 4 Department of Agro-Industrial Management and Technology, Federal University of Paraíba, Bananeiras, PB 58220-000, Brazil; prof.ufpb.anderson@gmail.com (A.F.V.); mariaufp@gmail.com (M.J.F.); 5 Department of Science and Technology, Federal Rural University of the Semi-Arid Region, Caraúbas, RN 59780-000, Brazil; rosilda.santos@ufersa.edu.br; 6 Teacher Training Center, Federal University of Campina Grande, Cajazeiras, PB 58900-000, Brazil; dallino@hotmail.com (F.D.R.); rodrigo.pre@ufcg.edu.br (R.A.A.S.)

**Keywords:** hydrocyclone, ceramic membrane, multiphase flow, water/oil separation, Ansys CFX^®^

## Abstract

This research aims to study the process of separating water contaminated with oil using a hydrocyclone with a porous wall (membrane), containing two tangential inlets and two concentric outlets (concentrate and permeate), at the base of the equipment. For the study, the computational fluid dynamics technique was used in a Eulerian–Eulerian approach to solve the mass and linear momentum conservation equations and the turbulence model. The effects of the concentration polarization layer thickness and membrane rejection coefficient on the permeate flow, hydrodynamic behavior of the fluids inside the hydrocyclone, and equipment performance were evaluated. Results of the velocity, transmembrane pressure and oil concentration profiles along the equipment, and hydrocyclone performance are presented and analyzed. The results confirmed the effect of the membrane rejection coefficient on the equipment performance and the high potential of the hydrocyclone with a porous wall to be used in the oil–water mixture separation.

## 1. Introduction

In the oil industry, it is common to use operations that lead to an increase in oil recovery efficiency. These processes use injection and production wells arranged to optimize oil production. In the injection wells, some fluids are usually introduced into the oil reservoir such as high-pressure water to maintain the system pressure and, consequently, move the oil towards the producing wells with greater efficiency. However, as the injected water and the water that exists in the reservoir itself have a viscosity that, in many situations, is lower than that of the oil, in general, regions of the porous medium (reservoir) that are not reached by the water appear. Therefore, the premature and increasing eruption of water in the producing wells is noticed, reducing the oil production and the system performance.

The water in the reservoir that is brought to the surface along with petroleum, gas, and other components during production activities is well known as produced water. This oil-contaminated water, in general, presents large volumes, but what to do with this oily water? The high volumes of produced water and the complexity of its composition have been of great concern to the oil industry due to technical and operational aspects and, mainly, environmental ones. As a consequence, the management of this water results in considerably high costs, which represent a significant percentage of production costs [1].

The produced water contains several parameters, which makes it potentially dangerous to the environment—for example, oil concentrations which vary from 50 to 5000 ppm; high salinity, such as sodium chloride (NaCl) at concentrations between 40,000 and 150,000 mg/L; and suspended solids content (TSS), ranging from 5 to 2000 ppm. Besides, dissolved microorganisms and gases, carbonic and hydrogen sulfide may be present [2].

Oil is among the various components present in the produced water, which has been of most concern to the sector due to the damage that this fluid can cause to the environment. This oil can be preset in produced water in three different ways:

(a) Free oil, dispersed in the form of drops with large diameters, above 100 μm, which, since it is already completely stratified in water, can be removed with relative ease, exclusively by physical processes.
(b) Soluble oil, which is composed of hydrocarbons less insoluble in water, such as benzene, toluene, ethylbenzene, and xylene (BTEX) and phenols.
(c) Emulsified oil, which exists in the dispersed form in small water drops, with a diameter varying from 20 to 100 μm.

Currently, the alternatives usually adopted by the oil industry for the destination of the produced water are disposal, injection, or reuse. However, the environmental effects caused by produced water when discarded are mostly evaluated by the toxicity and quantification of organic and inorganic compounds. Thus, due to the complexity of the quality of this water, the disposal site must be evaluated to verify the possible environmental impacts. Usually, this disposal is done only at sea, directly from offshore oil production platforms, or through submarine outfalls, in some onshore units.

However, the disposal of produced water must satisfy specific criteria defined by environmental regulatory agencies in order to minimize environmental impacts. In Brazil, the resolution established by the National Environment Council (CONAMA), nº 393/2007, provides for the continuous disposal of process or production water on offshore oil and natural gas platforms. In this resolution, a simple monthly arithmetic average concentration of oil and greases of up to 29 mg/L was established, which cannot exceed the daily limit of 42 mg/L [3]. To meet the environmental standards for disposal and/or the characteristics necessary for water reuse, the treatment can become a complex operation and dependent on highly efficient processes.

In the face of increasingly stringent requirements from regulatory agencies, meeting environmental safety standards has become one of the biggest challenges in the oil and gas sectors. Currently, several techniques are used to treat produced water, such as hydrocyclones and porous membranes (ceramic and polymeric).

According to the literature, the hydrocyclone has demonstrated great efficiency in the free oil/water separation process [4,5,6,7,8,9,10,11,12,13,14,15]. This equipment has a high processing capacity, requires little physical space for installation, is simple to operate, and requires low maintenance frequency. These advantages make this equipment economically viable for this type of activity, presenting a high benefit–cost ratio.

The performance of a hydrocyclone is affected by geometric (dimensions of the hydrocyclone, inlet diameter, and cylindrical and conical bodies, for example) and operational parameters (thermophysical properties of fluids and solids, the concentration of solids and liquids in the feed, intake pressure, and granulometry of the solid, for example) [16].

Despite its excellent characteristics, the hydrocyclone has demonstrated low efficiency when it comes to the separation of oil dispersed in water, especially when applied to cases where the oil droplets have small diameters and the oil concentration is low.

The use of membranes has been presented as a potential technological solution to the problem of oily effluent with small-diameter droplets. The membrane separation process uses the principle of filtration to separate immiscible solids or liquids and solutes that are dissolved, acting as a selective barrier, allowing the passage of certain components while preventing the passage of others. In this separation process, the feed stream is divided into two parts: the concentrate, which contains the contaminants initially present in the feed stream, and the permeate or purified, the fraction of liquid that has passed through the membrane.

The membranes have the characteristics of retaining oil droplets with a diameter below 10 µm, not requiring the use of chemical products, and of producing permeate with an oil concentration that meets the environmental legislation standards. For this reason, it has been the subject of studies by several researchers [17,18,19,20,21,22,23,24,25,26,27,28,29,30,31,32].

Despite presenting excellent characteristics, the membranes, when in use, present a continuous reduction in the permeate flow as a result of the accumulation of solute on the membrane surface (concentration polarization) and impregnation on the permeable surface (fouling or encrustation).

Therefore, depending on operational conditions, the mentioned technologies for the treatment of produced water are not always able to reach the required levels of performance when applied alone, requiring the combination of two or more treatment techniques [19].

In this sense, some studies using filtering hydrocyclones have been reported in the literature [33,34,35,36], in general applied in the mineral sector (solid–liquid separation), and there is little research related to the process of separating water contaminated by oil (liquid–liquid) [6,7,8,14,30,37,38].

Given the above, this work aims to study the process of separating produced water using a new hydrocyclone configuration, using the computational fluid dynamics technique. This equipment has the same operation principle as a conventional hydrocyclone, consisting of a porous wall (ceramic membrane) and two permeate and concentrate outlets at the bottom of the device, so it was called a filter cyclonic separator. In this way, it can be verified that it is a piece of equipment with an innovative design and differentiated geometry, constituting an attractive technology that is capable of significantly reducing the effect of the concentration polarization layer due to the swirl flow induced by the tangential entrances of the feed mixture. Besides, the equipment allows for additional removal of the permeate flow through the membrane pores and the formation of an oil core inside the equipment, which reduces the oil concentration in the vicinity of the membrane, positively affects the decline in permeate flow in this region, and increases equipment performance.

The authors expect to improve the proposed hydrocyclone to be applied in physical situations where the conventional hydrocyclone and membrane are not as efficient when operating alone in the treatment of water contaminated by oil.

## 2. Methodology

### 2.1. The Physical Problem and Geometry

**Figure 1:**
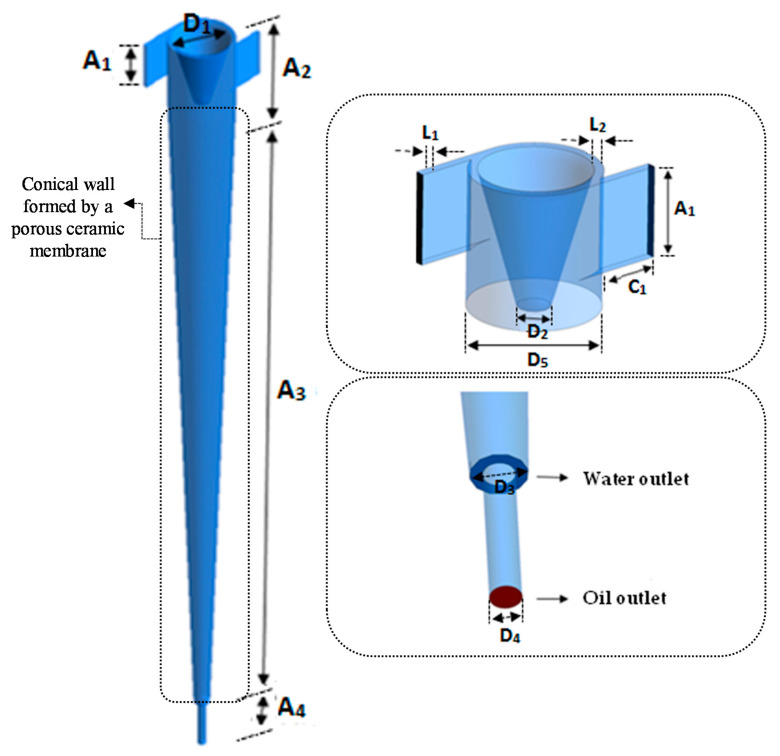
Schematic representation of the hydrocyclone of porous wall.

The study domain corresponds to a hydrocyclone formed by one main cone with two tangential inlets and two axial and concentric outlets, as pictured in Figure 1. In the vicinity of the tangential inlets, a tapered trunk was placed to direct the oil flowing inside the equipment to one of the axial outlets. Furthermore, the device has a conical wall that is formed by a porous ceramic membrane. The idea was to apply the new equipment for separating oil from produced water that originated from petroleum extraction. The dimensions of the separator are shown in Table 1.

**Figure 2:**
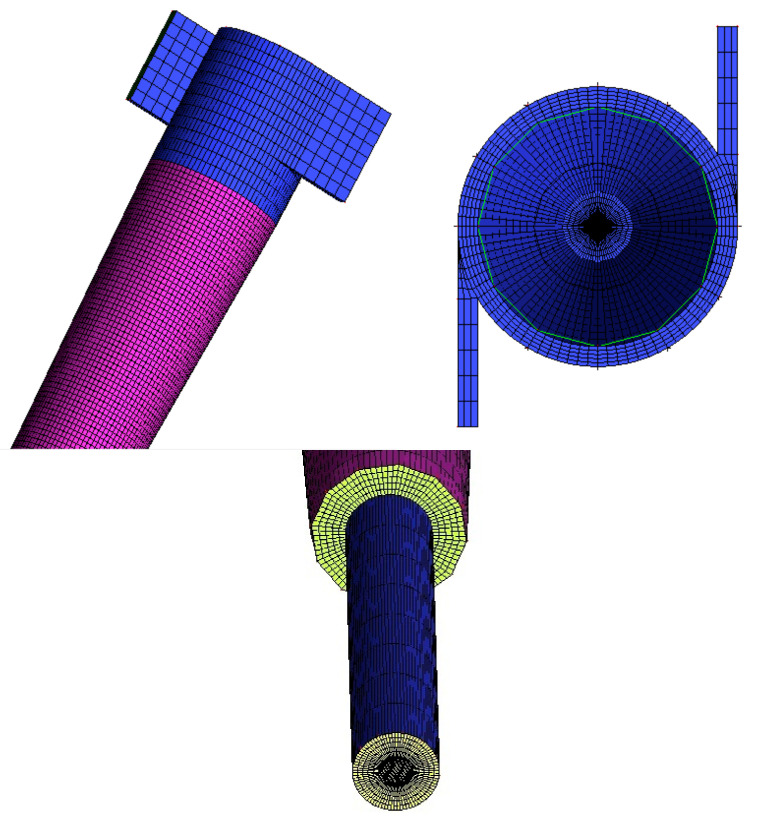
The numerical mesh used in this research.

To create the computational domain (mesh), Ansys ICEM CFD^®^ software (Ansys, Inc., Canonsburg, PA, USA) was used. To obtain coherent numerical results with lower computational effort, a mesh refining study was carried out using the mesh convergence index (ICM) method as proposed by Roache [39]. Figure 2 illustrates one of the meshes used in this study.

### 2.2. The Mathematical Model

The model used in this study corresponds to a generalization of the mass and linear momentum conservation equations (Navier–Stokes equations) by applying the Eulerian–Eulerian approach [7]. The following hypotheses were also considered by Cunha [20], Damak et al. [40], Souza [19], and Nunes [21]:

- Incompressible and Newtonian fluid with constant physical–chemical properties;
- Steady-state, turbulent and isothermal flow;
- Mass transfer, interfacial momentum, and mass source are disregarded;
- Non-drag interfacial forces such as lift forces, wall lubrication, virtual mass, turbulent dispersion, and solid pressure are neglected;
- The water stream is a multicomponent mixture of water and oil (solute);
- The walls are static and non-deformable;
- The porous wall (ceramic membrane) has constant permeability and porosity;
- The concentration polarization layer thickness is considered uniform and homogeneous;
- Chemical reaction or adsorption phenomena of the solute on the contact surface in the porous medium are neglected.

From the above-mentioned considerations, the following equations can be applied:

(a) Mass Conservation Equation:
  (1) $$\nabla \cdot \left(f_{\alpha }\rho_{\alpha }{\vec{U}}_{\alpha }\right)=0$$
  where the Greek sub-index α represents the phase involved in the two-phase water/oil mixture; f, ρ, and e $\vec{U}$ are the volume fraction, density, and velocity vector, respectively.
(b) Momentum Conservation Equation:
  (2) $$\nabla \cdot \left[f_{\alpha }\left(\rho_{\alpha }{\vec{U}}_{\alpha }⊗{\vec{U}}_{\alpha }\right)\right]=-f_{\alpha }\nabla p_{\alpha }+\nabla .\left\{f_{\alpha }\mu_{ef}\left[\nabla {\vec{U}}_{\alpha }+{\left(\nabla {\vec{U}}_{\alpha }\right)}^{T}\right]\right\}+{\vec{M}}_{\alpha },$$
  where $p_{\alpha }$ is the pressure of phase α and $M_{\alpha \;}$ describes the drag force per unit volume on phase α due to the interaction with phase *β*, being defined by:
  (3) $${\vec{M}}_{\alpha }\;=\;\vec{M_{\alpha \beta }^{D}}=C_{\alpha \beta }^{\left(d\right)}\left({\vec{U}}_{\beta }-{\vec{U}}_{\alpha }\right),$$
  where $C_{\alpha \beta }^{\left(d\right)}$ is the dimensionless drag coefficient given by:
  (4) $$C_{\alpha \beta }^{\left(d\right)}=\;\frac{3}{4}\frac{C_{D}}{d_{p}}\;f_{\beta }\rho_{\alpha }\left|{\vec{U}}_{\beta }-{\vec{U}}_{\alpha }\right|,$$
  where *C_D_* = 0.44 represents the drag coefficient and d_p_ represents the particle diameter. The term $\nabla .\left\{f_{\alpha }\mu_{ef}\left[\nabla {\vec{U}}_{\alpha }+{\left(\nabla {\vec{U}}_{\alpha }\right)}^{T}\right]\right\}$ is the momentum transfer induced by the interfacial mass transfer, and $\mu_{ef}$ is the effective viscosity, defined by:
  (5) $$\mu_{ef}=\mu +\mu_{t},$$
  where μ is the dynamic viscosity and $\mu_{t}$ is the turbulent viscosity. The turbulent viscosity is a function of turbulent flow intensity and is unknown. It is necessary to use models to predict their values.
  The following mass transport equation was used:
  (6) $$\vec{U}.\nabla C=\;D_{AB}\;\nabla^{2}C$$
  where C is the solute concentration and $D_{AB}=1.12\times 10^{-8}\;m^{2}/s$ is the mass diffusion coefficient, defined as:
  (7) $$D_{AB}=\frac{\mu }{\rho S_{C}},$$
  where *μ* is the dynamic viscosity and $S_{C}$ corresponds to the Schmidt number.
(c) Turbulence model
  The turbulence model chosen for the continuous phase was the well-known SST (Shear Stress Transport) turbulence model. In this model, close to the fluid/membrane interface, the k−ω model is applied, and according to the need, where this model does not show good results, the k−ε model is applied. The choice of the model was because the cases studied have more pronounced pressure and concentration gradients near the fluid/membrane interface.
(d) Separation efficiency
  To evaluate the efficiency of water/oil separation, the total efficiency was used, which can be calculated as the ratio between the mass flow rate of oil droplets of a given size $\left(d\right)$ found in the overflow, $W_{go}\left(d\right)$, and the mass flow rate of the oil in the feed, $W_{g}\left(d\right)$, given by the equation:
  (8) $$G\left(d\right)=100\times \;\frac{W_{go}\left(d\right)}{W_{g}\left(d\right)}\;$$
  To verify only the amount of oil collected in the overflow by the exclusive effect of the hydrocyclone centrifugal field, the reduced separation efficiency $\left(G^{\prime }\right)$ was considered as follows:
  (9) $$G^{\prime }=\frac{\left(G-\;R_{L}\right)}{\left(1-\;R_{L}\right)},$$
  where $R_{L}$ is a parameter that relates the mass flow rate of water collected in the overflow $\left(W_{lo}\right)$ and the mass flow rate of water fed in the hydrocyclone $\left(W_{l}\right)$, called the liquid ratio:
  (10) $$R_{L}=\;\frac{W_{lo}\left(d\right)}{W_{l}\left(d\right)}.$$

Details about the efficiency formulation given by equations 8, 9, and 10 can be found in the literature [16,21,41].

### 2.3. Boundary Conditions

For the solution of the governing equation, different conditions at the domain boundaries were established: Input, Porous wall (permeate), Non-porous walls, and Outputs (concentrated and diluted). Details about this topic can be found in Nunes et al. [37].

### 2.4. Process Parameters and Evaluated Cases

The hydrocyclone was evaluated through numerical simulations using Ansys CFX 15.0 software. The simulations were performed using a convergence criterion of $10^{-7}\mathrm{kg}/s$ in (root mean square) for all unknown variables. Table 2 summarizes the parameters of the fluid phases and membrane used in the model. The solute concentration was inserted into the software as a mass fraction and the interfacial tension of 0.01 N/m  was considered.

Table 3 illustrates the input data of the different cases studied in this research. Case 1 was used in the study of mesh refining. Cases 2 and 3, on the other hand, were used to evaluate the effect of the polarization layer thickness, and Cases 3–7 were used to evaluate the effect of the membrane rejection coefficient on the fluid dynamic behavior and separation performance of the hydrocyclone. Membrane rejection coefficients were established arbitrarily while the polarization layer thickness was calculated based on the literature [20,21,22,40].

## 3. Results and Discussion

### 3.1. Mesh Refinement Study

The mesh quality analysis was performed using the mesh convergence index method (ICM) for Case 1 (Table 3). For that, three meshes (M1, M2, and M3) of the hydrocyclone were generated with different refinement degrees. In this study, the refining ratios of 1.6 and 1.8 between meshes M1 and M2 and between meshes M2 and M3, respectively, were used. These values are within the range proposed by Roache [39]. Table 4 summarizes the number of elements and the simulation time obtained with the different meshes.

**Figure 3:**
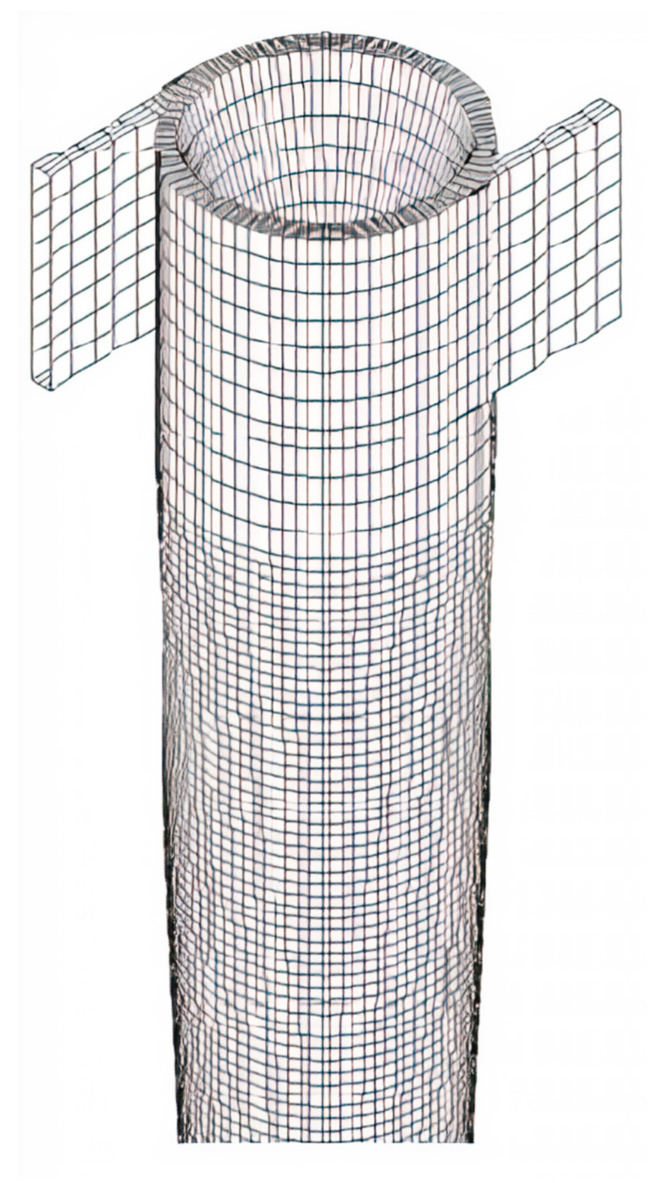
Details of the numerical mesh M1 used in the simulations.

Details of the most refined mesh are shown in Figure 3. It is important to state that refinement was carried out in the conical region of the study domain due to the possibility of the presence of high concentration gradients in that region.

**Figure 4:**
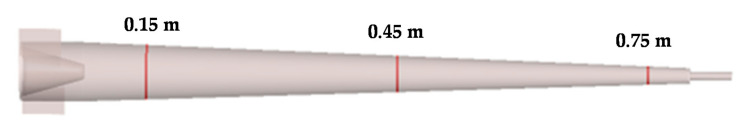
Location of the study lines carried out along the cyclonic separator.

To analyze the behavior of the hydrodynamic variables, horizontal lines were drawn in three axial positions along the length of the computational domain (y = 0.15, 0.45, and 0.75 m), as shown in Figure 4.

Table 5 and Table 6 show the results of the convergence study for the oil mass flow rate at the oil outlet and the water mass flow rate at the oil outlet, respectively, for different mesh sizes, M1, M2, and M3, in comparison with the extrapolated solution, indicated by Me. In these cases, it is possible to observe a reduction in the convergence condition since $ICM_{21}<\;ICM_{32}$, which indicates that the dependence of the results on the size of the elements of the mesh has been reduced and approaches the solution independent of the mesh. Besides, the values of $ICM_{21}\;$ and $ICM_{32}$ are within the 10% limit, as reported by Karatekin [42]. According to the criteria established by Paudel and Saenger [43], the value of the C parameter indicates monotonic convergence of the solution. Finally, it is possible to observe that the extrapolated solution is close to the exact solution for this variable, due to the proximity of the values of $ICM_{32}\;$ and $r^{p}ICM_{21}$. These results indicate that the more refined the mesh, the more the solution approaches the asymptotic value of the extrapolated solution, with the M1 mesh solution being the closest. The extrapolated solution represents an estimation of the exact solution for the studied variable. It is also possible to observe an increase in the oil mass flow rate at the oil outlet and a decrease in the oil mass flow rate at the water outlet, a fact that leads to better separation efficiency results, as we will see below.

Table 7 presents the results of the convergence study for reduced separation efficiency. There is a reduction in the convergence condition since $\;ICM_{21}<\;ICM_{32}$, which indicates that the dependence of the results on the size of the elements of the mesh has been reduced and approaches an independent solution of the mesh. Besides, the values of $ICM_{21}\;$ and $ICM_{32}$ are within the 10% limit as reported by Karatekin [42].

Similar to what was observed in the ICM study of the conventional cyclonic separator, these results confirm what has already been observed in the oil mass flow rate at the water outlet and in the water mass flow at the oil outlet, which indicates that the more refined mesh is that which presents a greater separation efficiency, and consequently, this value approaches the asymptotic value of the extrapolated solution.

**Figure 5:**
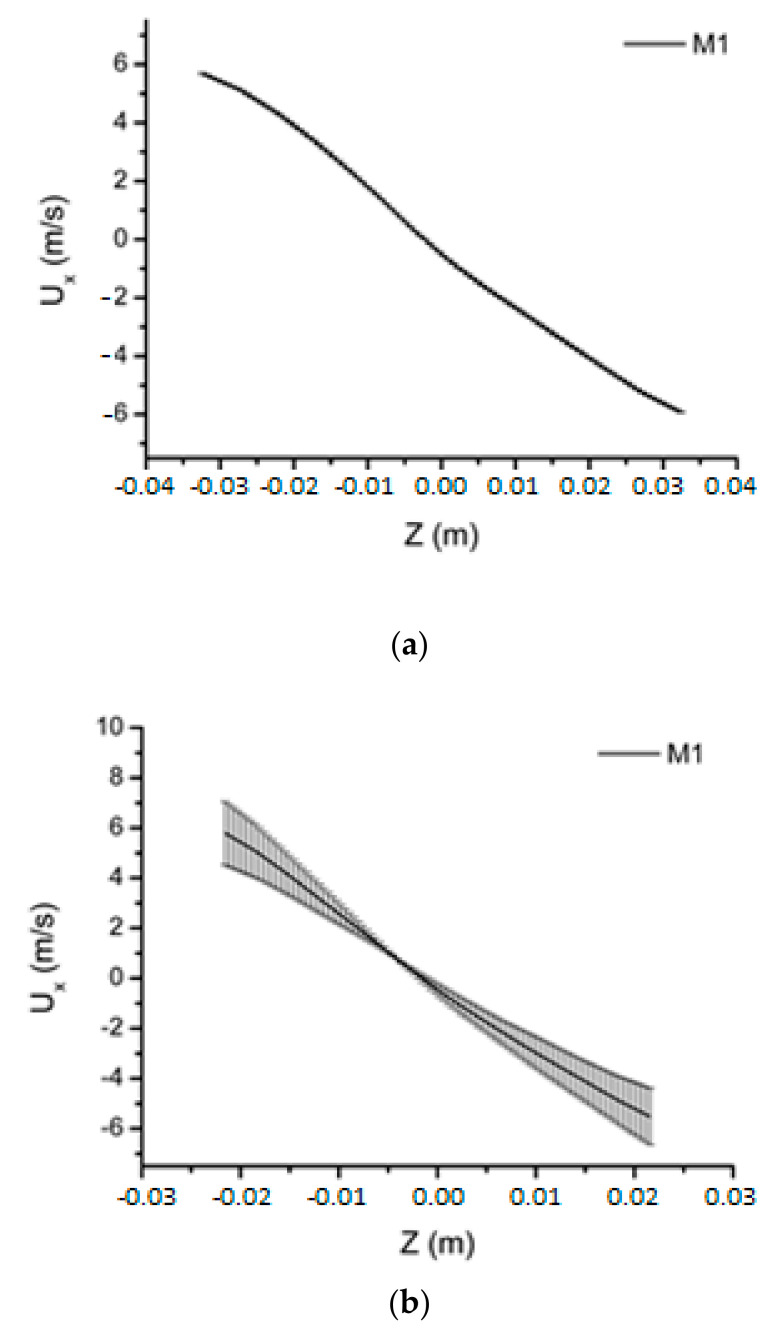

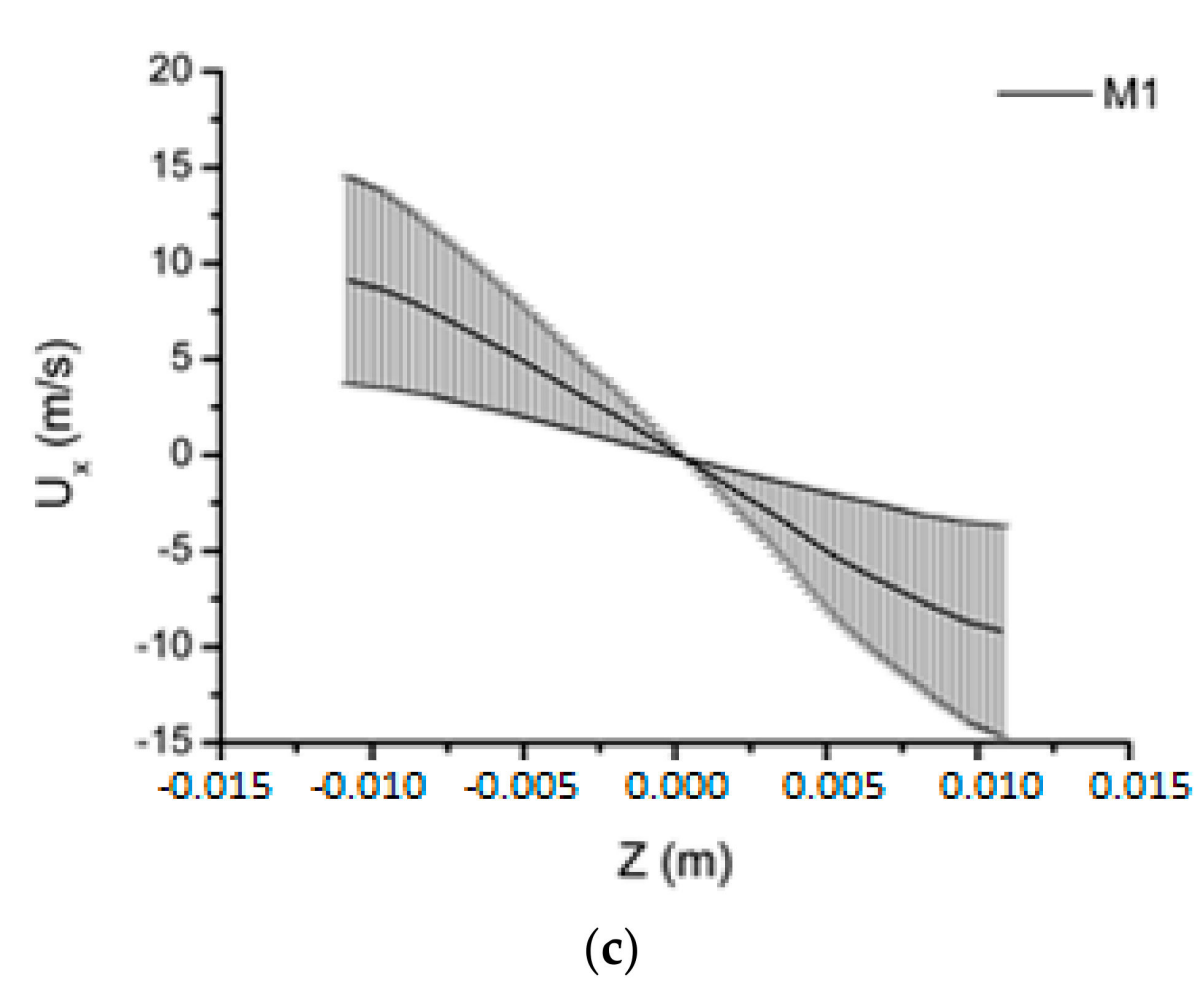
Water tangential velocity in the filtering cyclonic separator for the M1 mesh with *ICM_21_* in the form of an error bar. (**a**) y = 0.15 m; (**b**) y = 0.45 m, and (**c**) y = 0.75 m.

**Figure 6:**
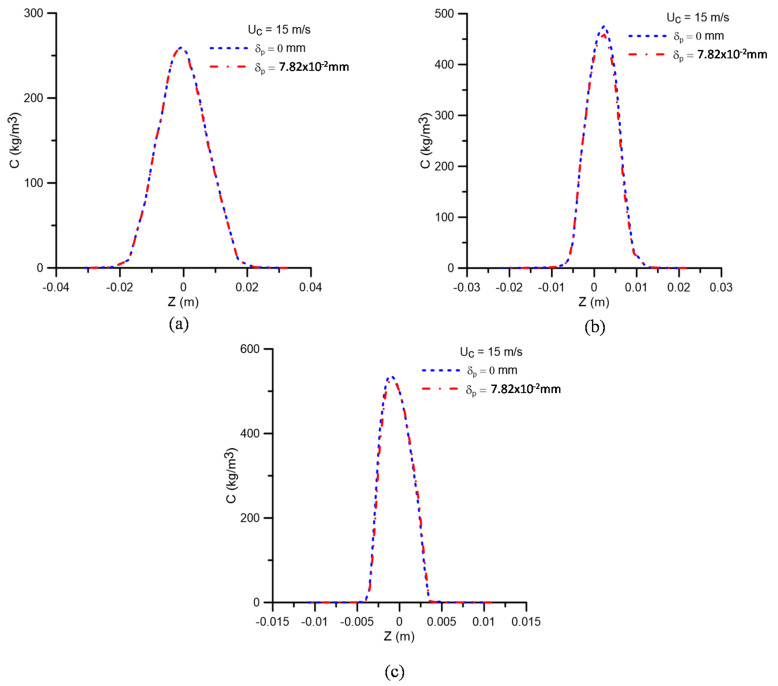
Oil concentration profiles in the positions: (**a**) y = 0.15 m; (**b**) y = 0.45 m, and (**c**) y = 0.75 m, with $\delta_{p}$ = 0, $\delta_{p\;}$= 7.82 × 10^−2^ mm, and f_o_ = 5%.

Figure 5 shows the results of the water tangential velocity in the positions y = 0.15 m, y = 0.45 m, and y = 0.75 m for the M1 mesh with *ICM_21_* plotted in the form of error bars. For the positions analyzed, the mean p-value ranged from 0.71 to 1.34. The average value of *ICM_21_* varied between 6.3% and 9.63%. When compared to Figure 6, which shows the water tangential velocity of the M1 mesh with *ICM_21_* presented in the form of error bars of the conventional cyclonic separator, a change in the error bars is observed, a fact that can be explained because the filtering wall modifies the behavior of the tangential velocity, as will be described in a later section.

According to the analysis of the meshes, in the conventional cyclonic separator and the cyclonic filter separator, it can be said that in both cases, the more refined mesh (M1), which contains approximately 337,000 elements, is within the asymptotic range since *ICM_21_* < *ICM_32_* and the points were below 10%, a limit determined in the studies by Karatekin [42]. Thus, it can be noticed that the M1 mesh presented a solution for the studied variables, totally independent of the mesh.

### 3.2. Hydrodynamic and Performance Analyses

Figure 6 illustrates the oil concentration profile at different axial positions along the hydrocyclone for two values of concentration polarization layer thickness ($\delta_{p}$ = 7.82 × 10^−2^ mm and $\delta_{p}$ = 0 mm). Analyzing this figure, it can be seen that by setting the feed velocity at 15 m/s and the oil volume fraction at 5%, the oil concentration profiles are not significantly affected in the central region of the equipment and on the membrane surface when considering the effect of the concentration polarization layer thickness. This can be explained by two reasons: (a) because smaller oil volume fractions produce higher axial components of velocities, and (b) due to increased shear on the membrane surface, favoring oil transport from the membrane surface towards the oil core in the hydrocyclone, which, in turn, can be explained by the difference in the density of the fluids and the effect of the forces (centrifugal, gravitational, drag, and centripetal) acting on the fluids. Similar behavior was observed by Barbosa [6], Luna [7], and Zimmermann [44] using conventional and/or modified hydrocyclones.

**Figure 7:**
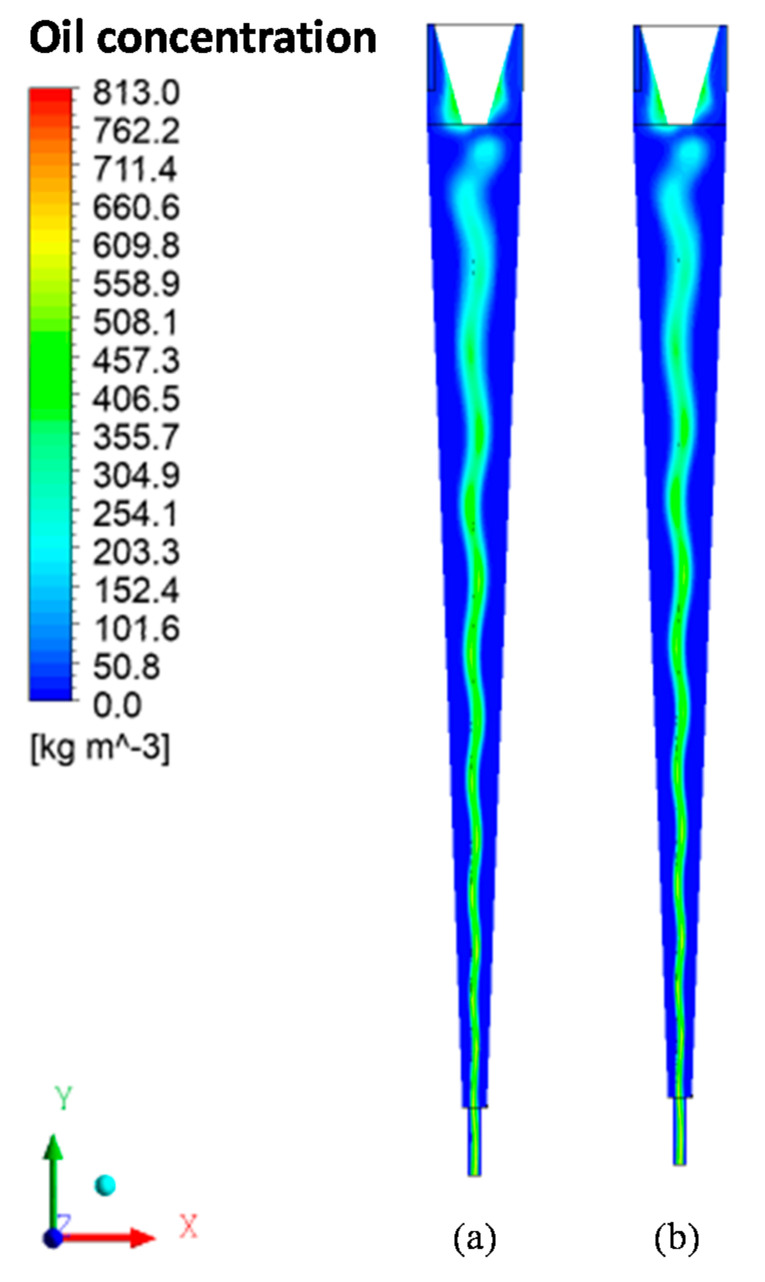
Oil concentration fields in the xy plane for the case f_o_ = 5% and feed velocity of 15 m/s with (**a**) $\delta_{p}$ = 0 mm, and (**b**) $\delta_{p}$ = 7.82 × 10^−2^ mm.

Figure 7 illustrates the oil concentration fields in the xy plane inside the hydrocyclone for a 5% oil volume fraction and two polarization layer thicknesses ($\delta_{p}$ = 7.82 × 10^−2^ mm e $\delta_{p}$ = 0 mm). Analyzing this figure, it can be noticed that there is an increase in the oil concentration in the center of the equipment, forming an oil core. The oil core tends to expand and a small fraction of oil tends to get closer to the conical wall of the cyclonic separator. This fact is due to the centrifugal forces that act with greater intensity in the denser phase, water, and provide the dragging of oil droplets, directing them to the oil core. In all cases, the oil core remains stable in the central region of the filtering cyclonic separator. These results indicate that the concentration field is not affected when considering the effect of the polarized layer.

**Figure 8:**
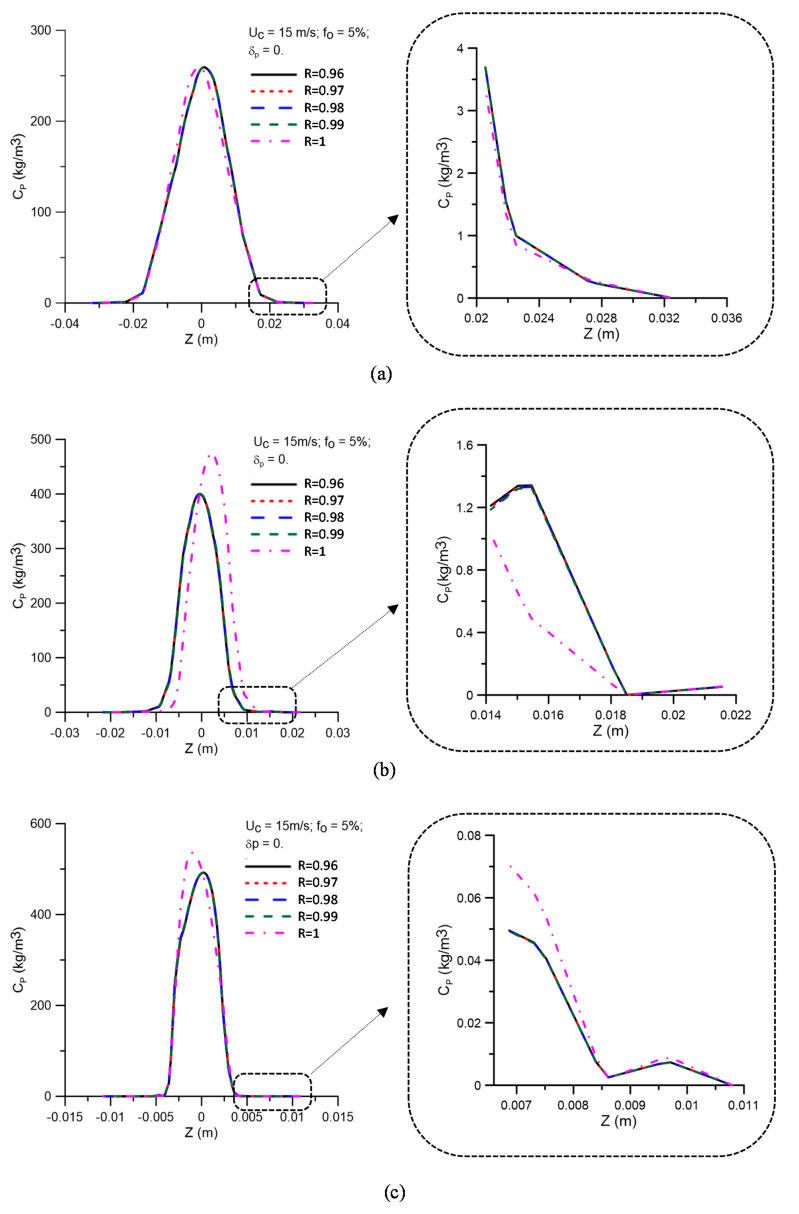
Oil concentration profiles for different values of the membrane rejection coefficient in the positions: (**a**) y = 0.15 m; (**b**) y = 0.45 m, and (**c**) y = 0.75 m, with $\delta_{p\;}$ = 0 mm.

**Figure 9:**
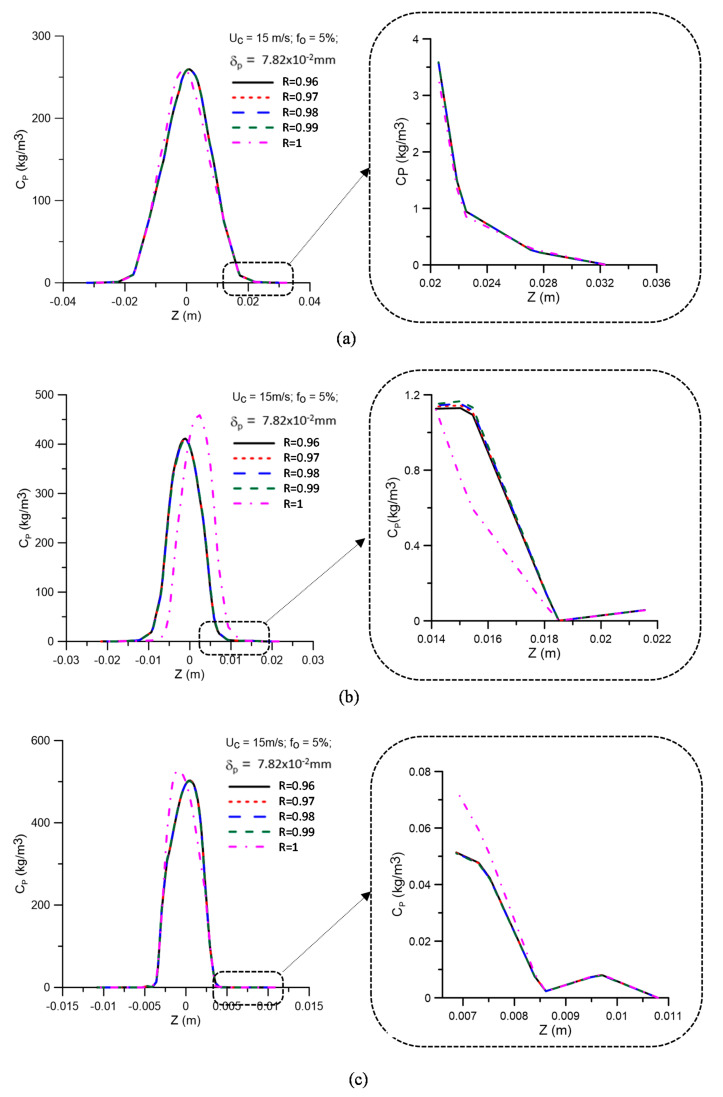
Oil concentration profiles for different values of the membrane rejection coefficient in the positions: (**a**) y = 0.15 m; (**b**) y = 0.45 m, and (**c**) y = 0.75 m, with $\delta_{p}$ = 7.82 × 10^−2^ mm.

Figure 8 shows the oil concentration profiles for different values of the membrane rejection coefficient (R = 0.96, 0.97, 0.98, 0.99, and 1.00), represented as a function of the transverse position on the z-axis, at positions y = 0.15, 0.45, and 0.75 m, as illustrated in Figure 4. Details (enlargement) of the oil concentration profiles in the vicinity of the equipment’s porous wall (ceramic membrane) are also illustrated in these figures. As already observed by Magalhães [45], there is a similar behavior between the concentration profiles in the central region and the region close to the porous hydrocyclone wall (membrane) for the different rejection coefficients (R = 0.96, 0.97, 0.98, and 0.99). However, there is a variation in the behavior of the oil concentration in the central region and close to the membrane surface, as seen in Figure 8b,c for the case with the maximum rejection coefficient (R = 1.00). A similar fact can also be observed in Figure 9, which presents the oil concentration profiles for different values of the membrane rejection coefficient, represented as a function of the transverse position on the z-axis, at positions y = 0.15, 0.45, and 0.75 m, when considering the effect of the polarization layer thickness. The analysis of these figures shows that the oil concentration profiles are not significantly changed when considering the effect of the concentration polarization layer.

**Figure 10:**
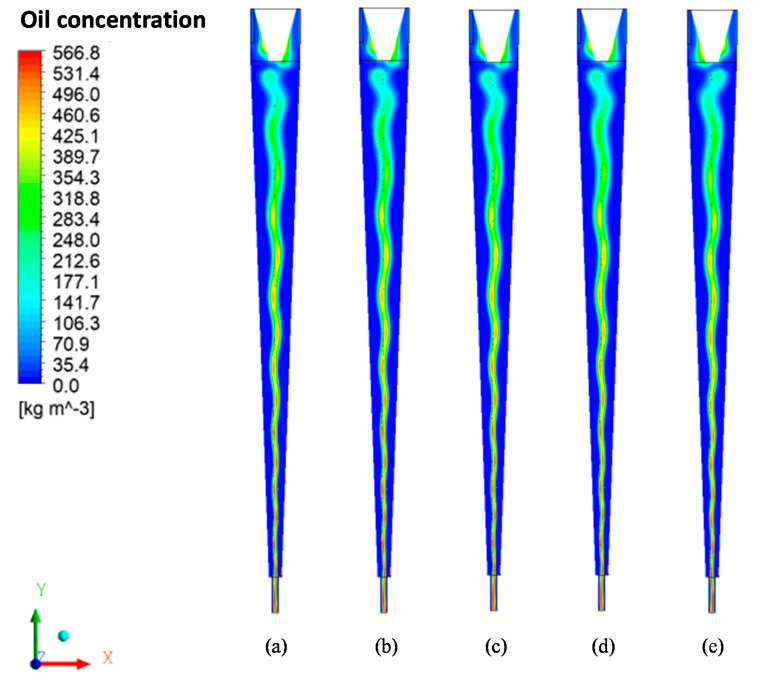
Oil concentration fields for different values of the membrane rejection coefficient: (**a**) R = 0.96; (**b**) R = 0.97; (**c**) R = 0.98; (**d**) R = 0.99, and (**e**) R = 1.00, with $\delta_{p}$ = 7.82 × 10^−2^ mm.

Figure 10 presents the oil concentration fields, in the xy plane, for different values of the solute rejection coefficient by the membrane (R = 0.96, 0.97, 0.98, 0.99, and 1.00). Analyzing this figure, it is possible to observe, in all cases, the formation and stability of the oil core in the central region of the filtering cyclonic separator. Besides, it is noted that the variation of the solute rejection coefficient by the membrane does not significantly affect the hydrodynamic behavior of the oil core. This fact can be explained by the turbulence induced by the tangential entrances and, thus, the predominance of the tangential component of velocity, concerning the axial component of velocity, inside the filtering cyclonic separator.

**Figure 11:**
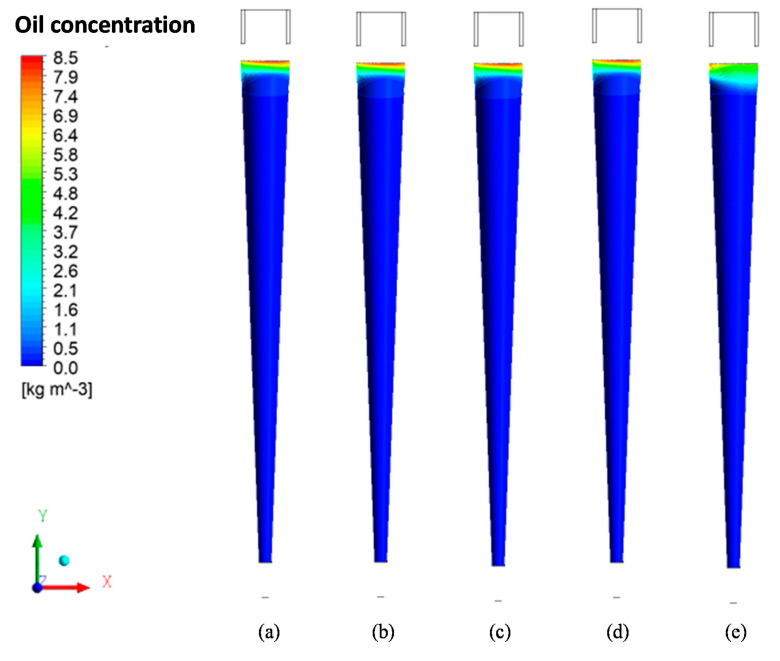
Oil concentration fields on the membrane wall for different values of the membrane rejection coefficient: (**a**) R = 0.96; (**b**) R = 0.97; (**c**) R = 0.98; (**d**) R = 0.99, and (**e**) R = 1.00, with $\delta_{p}$ = 7.82 × 10^−2^ mm.

Figure 11 shows the oil concentration fields on the membrane surface for different values of the solute rejection coefficient by the membrane (R = 0.96, 0.97, 0.98, 0.99, and 1.00). An analysis of this figure shows, in all simulated cases, that the membrane tends to concentrate oil in the upper region near the fluid inlet. This behavior can be explained by the high turbulence in the regions close to the conical trunk that causes a local mixture of water with the oil droplets, as can be seen in Figure 10, where the oil core has not yet formed.

**Figure 12:**
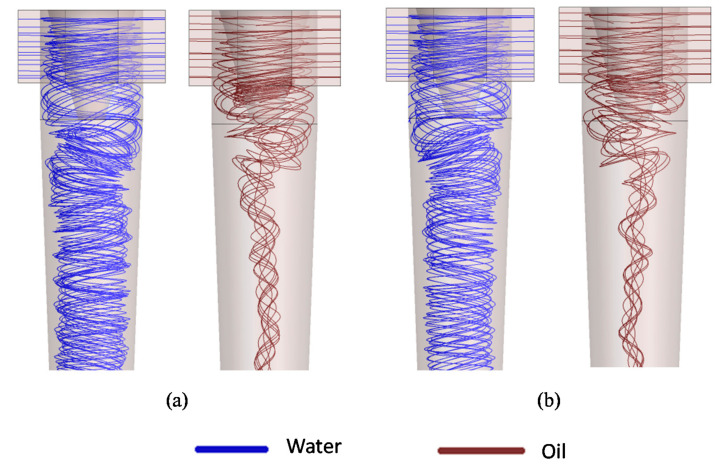
Water and oil streamlines inside the filtering cyclonic separator: (**a**) R = 0.96 and (**b**) R = 1.00, with $\delta_{p}$ = 7.82 × 10^−2^ mm.

Figure 12 shows the behavior of the streamlines in the region close to the tapered trunk and entries of the filtering cyclonic separator considering two rejection coefficients, R = 0.96 and 1.00. When observing this figure, a small variation in the behavior of the water and oil streams can be noticed, indicating the low influence of the rejection coefficient on the fluid dynamic behavior inside the filtering hydrocyclone.

The behavior of the fluids inside the equipment, observed in Figure 10, Figure 11 and Figure 12, indicates that there is a reduction in oil close to the membrane, which leads to an increase in the oil concentration in the central region. As the fluids (water and oil) move away from the tapered trunk, the angular momentum exceeds the axial momentum; that is, the tangential velocity component is greater than the axial velocity component, thus providing a greater shear close to the membrane surface and conducting the oil particles towards the oil core, as shown in Figure 10. This favors the permeate flow through the membrane and its useful life since this turbulence tends to clean the membrane surface continuously, minimizing the flow of oil into the membrane and increasing membrane performance.

**Figure 13:**
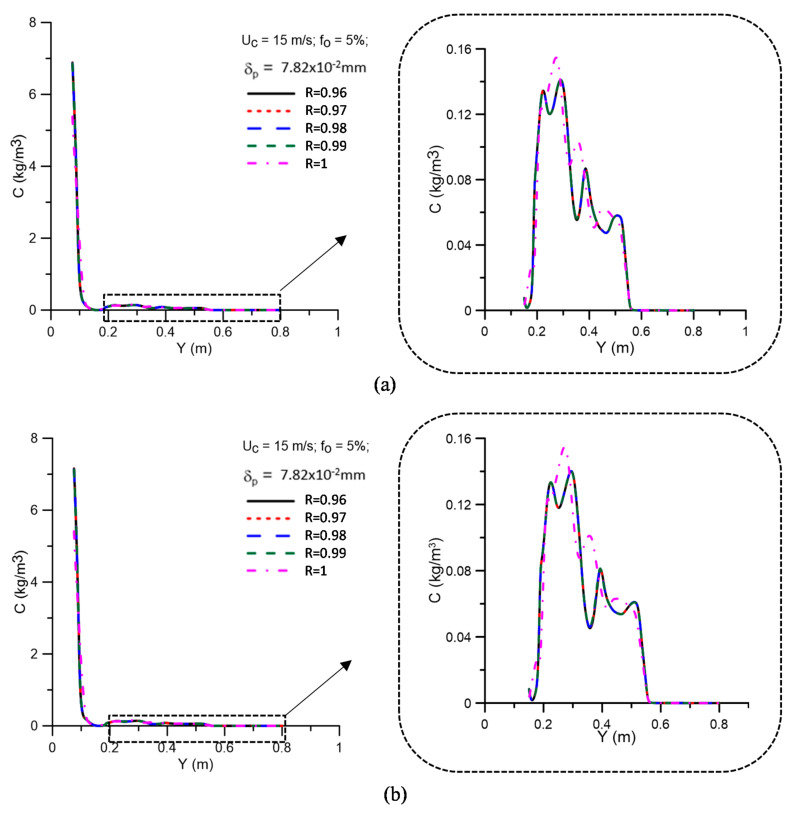
Oil concentration profiles as a function of the longitudinal position in the vicinity of the membrane for different values of the rejection coefficient, with (**a**) $\delta_{p}$ = 0 mm and (**b**) $\delta_{p}$ = 7.82 × 10^−2^ mm.

Figure 13 shows the oil concentration profiles as a function of the longitudinal position in the vicinity of the membrane for different values of the membrane rejection coefficient, with and without the effect of the polarized layer. Upon examining this figure, it can be seen that in both cases, when the selective capacity of the membrane is varied, the oil concentration on the membrane surface is not significantly altered; however, there is a higher oil concentration on the surface when the rejection coefficient is at a maximum (R = 1.00). This fact causes greater resistance to the permeate flow through the membrane.

**Figure 14:**
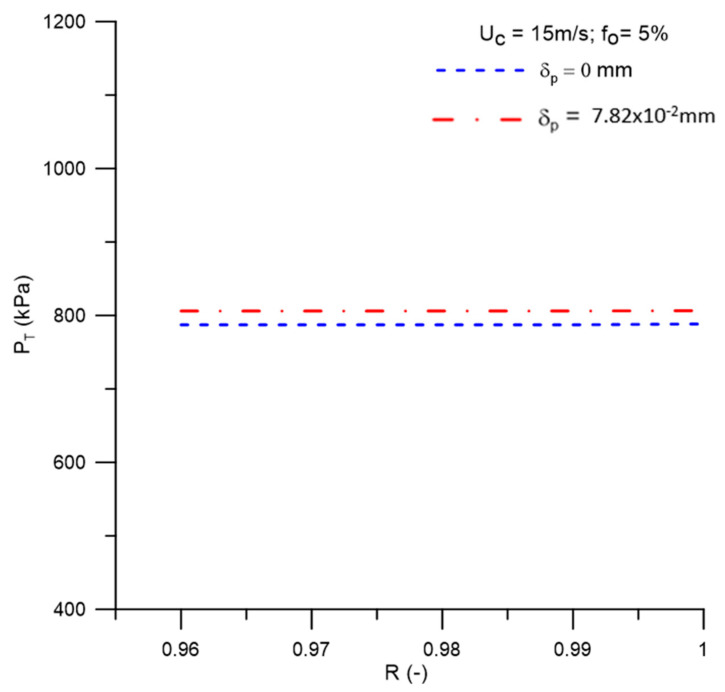
Transmembrane pressure behavior as a function of the membrane rejection coefficient.

Figure 14 shows the values of the transmembrane pressure as a function of the membrane rejection coefficient, obtained when considering, or not, the effect of the concentration polarization layer. It is noticed that when increasing the selective capacity of the membrane, the transmembrane pressure remains practically constant, with a slight increase for the case where $\delta_{p}$ = 7.82 × 10^−2^ mm in relation to $\delta_{p}$ = 0 mm. The approximately constant behavior of the transmembrane pressure with the increase in the rejection coefficient was also observed by Paris et al. [46] and Pradanos et al. [47] when studying the mass transfer coefficient and the rejection coefficient of an asymmetric ultrafiltration membrane using crossflow.

**Figure 15:**
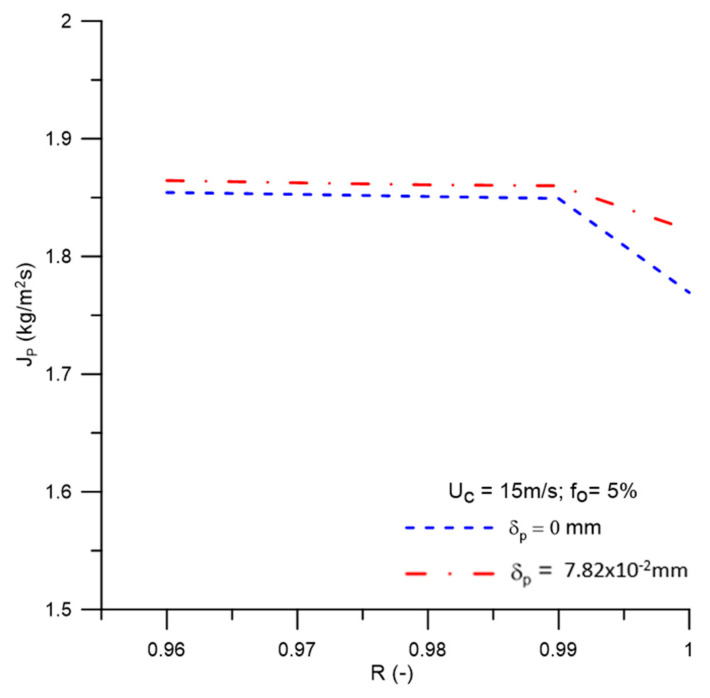
Permeate flow behavior as a function of the membrane rejection coefficient.

**Figure 16:**
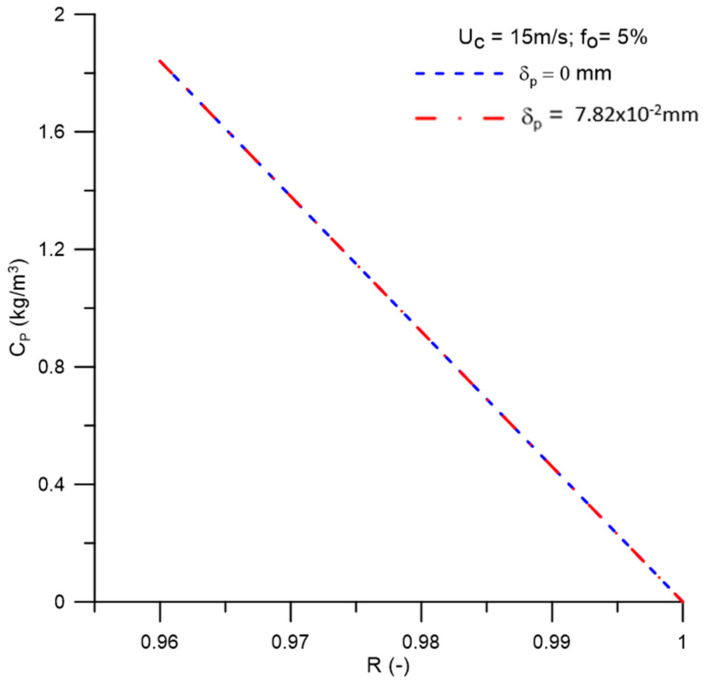
Oil concentration behavior in the permeate for different values of the membrane rejection coefficient.

Figure 15 shows the behavior of the permeate flow as a function of the membrane rejection coefficient with different concentration polarization layer thicknesses, $\delta_{p}$ = 0 mm and $\delta_{p}$ = 7.82 × 10^−2^ mm. In this figure, it can be seen that for values of the rejection coefficient up to 0.99, the permeate flow remained practically constant, showing a decrease for the interval 0.99 ≤ R ≤ 1. This is due to the increase in the transport resistance of the water caused by the oil concentration on the membrane surface, which offers greater resistance to the permeate flow. Similar behavior was reported by Habert et al. [48] when evaluating membrane separation processes. Besides, it can be seen that for lower membrane rejection coefficients, there is a greater permeate flow. However, when looking at Figure 16, which represents the oil concentration in the permeate for different values of the membrane rejection coefficient, there is an increase in the oil concentration in the permeate with the reduction in the rejection coefficient, as expected.

**Figure 17:**
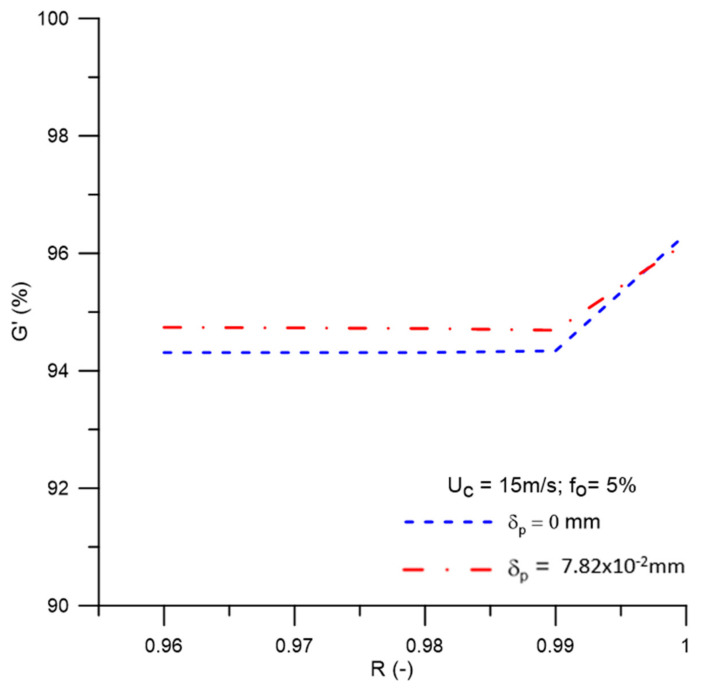
Reduced efficiency behavior of the hydrocyclone for different values of the membrane rejection coefficient.

Figure 17 shows the reduced efficiency of the filtering cyclonic separator for different values of the selective membrane capacity, $\delta_{p}$ = 0 mm and $\delta_{p}$ = 7.82 × 10^−2^ mm. Upon analyzing this figure, a small variation in the reduced efficiency can be seen (around G’ = 94%) by increasing the membrane rejection coefficient in the range 0.96 ≤ R ≤ 0.99. The highest reduced efficiency was obtained for the maximum rejection coefficient (R = 1.00). A similar fact can be observed when analyzing the reduced efficiency considering $\delta_{p}$ = 7.82 × 10^−2^ mm. This is because the rejection coefficient, when elevated, reduces the permeate flow and increases the resistance of the fluid to flow through the pores of the membrane. As a consequence, there is a smaller amount of oil transported by convection to the membrane wall and through the permeate flow, thus reducing the oil concentration in the permeate and increasing the separation efficiency.

## 4. Conclusions

Based on the predicted results, it can be concluded that:

(a) The proposed mathematical modeling successfully correctly described the multiphase flow behavior within a hydrocyclone with a porous membrane wall.
(b) The hydrocycloning process assisted by the filtration process was capable of altering the performance of the separation equipment.
(c) The hydrocyclone tends to concentrate the oil in the central region throughout the flow. However, for high oil concentrations, the core expanded and the oil particles approached the porous membrane wall of the device.
(d) The oil concentration profile is not significantly affected when considering the effect of the concentration polarization layer thickness in the central region of the equipment on the membrane surface, in the range of 0 ≤ $\delta_{p}$ ≤ 7.82 × 10^−2^ mm.
(e) The solute rejection coefficient by the membrane does not significantly affect the hydrodynamic behavior of the fluids inside the filtering cyclonic separator in the range 0.96 ≤ R ≤ 1.00. However, for higher membrane rejection coefficients (R = 1.00), there is a decrease in the mass flow rate of the permeate (1.822 kg/m^2^s), with minimal oil concentration in this mixture (0.0 kg/m^3^).
(f) When the selective capacity of the membrane is increased, the transmembrane pressure remains practically constant (≈787.2 kPa), with a slight increase (≈806.0 kPa) when considering the concentration polarization layer thickness ($\delta_{p}$ = 7.82 × 10^−2^ mm).
(g) The efficiency of the hydrocyclone remained approximately constant in the range of 0.96 ≤ R ≤ 0.99 (≈94.3%), rising from this point until reaching a value of 96.33% for R = 1.00. This parameter was higher when the concentration polarization layer thickness was varied from $\delta_{p}$ = 0 mm to $\delta_{p}$ = 7.82 × 10^−2^ mm, except for R = 1.00, where an inverse behavior was verified (≈96.18%).

## Figures and Tables

**Table 1:** Geometrical parameters of the hydrocyclone.

Tangential inlets (mm)	Height (A_1_)	50
	Length (C_1_)	50
	Width (L_1_)	5
Upper conical part (mm)	Height (A_2_)	75
	Width (L_2_)	5
	Top Diameter (D_1_)	65
	Bottom Diameter (D_2_)	18
Cylindrical section (mm)	Height (A_2_)	75
	Diameter (D_5_)	70
Conical section (mm)	Height (A_3_)	725
Annular outlet (mm)	Diameter (D_3_)	18
Tubular outlet (mm)	Diameter (D_4_)	10
	Height (A_4_)	50

**Table 2:** Physical, chemical, and geometrical parameters of the membrane and fluids phases (T = 293.15 K).

Membrane	Permeability	$1.39\;\times \;10^{-15}{\;m}^{2}$ [21]
	Polarization layer thickness	0.255 mm [21]
Water	Density	$997\;\mathrm{kg}/m^{3}$
	Viscosity	$8.889\times 10^{-4}\;\mathrm{Pa}.s$
	Molar mass	18.05 kg/kmol
Oil	Density	$868.7\;\mathrm{kg}/m^{3}$
	Viscosity	0.985 Pa.s
	Molar mass	873 kg/kmol
	Average oil drop diameter	0.1 mm

**Table 3:** Operational parameters used in the simulations.

Case	Input Velocity (m/s)	Oil Volume Fraction (%)	Membrane Rejection Coefficient R (-)	Polarization Layer Thickness (mm)
1	5	5.0	1	0
2	15	5	1	0
3	15	5	1	7.82 × 10^−2^
4	5	5	0.99	7.82 × 10^−2^
5	15	5	0.98	7.82 × 10^−2^
6	15	5	0.97	7.82 × 10^−2^
7	5	5	0.96	7.82 × 10^−2^

**Table 4:** Mesh data obtained in the convergence index analysis.

Mesh	Number of Elements	Simulation Time
M1	337,360	3 d 8 h 4′2″
M2	71,352	21 h 38′40″
M3	10,571	17′4″

**Table 5:** Results of the study of mesh convergence for the oil mass flow rate at the oil outlet.

M1 $\left(kg/s\right)$	M2 $\left(kg/s\right)$	M3 $\left(kg/s\right)$	p	$\phi_{ext}^{21}\;(\mathrm{Me})$ $\left(kg/s\right)$	$ICM_{21}$	$ICM_{32}$	$r^{p}ICM_{21}$
0.106	0.103	0.101	1.36	0.108	0.641	1.683	1.298

**Table 6:** Results of the study of mesh convergence for the water mass flow rate at the oil outlet.

M1 $\left(kg/s\right)$	M2 $\left(kg/s\right)$	M3 $\left(kg/s\right)$	p	$\phi_{ext}^{21}\;(\mathrm{Me})$ $\left(kg/s\right)$	$ICM_{21}$	$ICM_{32}$	C	$r^{p}ICM_{21}$
0.626	0.632	0.653	1.96	0.622	0.291	1.611	0.32	0.805

**Table 7:** Results of the study of mesh convergence for reduced efficiency.

$M1\;\left(\%\right)$	$M2\;\left(\%\right)$	$M3\;\left(\%\right)$	p	$\phi_{ext}^{21}$ $\left(\%\right)$	$ICM_{21}$	$ICM_{32}$
97.16	94.15	91.70	1.24	100	0.86	2.68
